# Ensuring Explainability and Dimensionality Reduction in a Multidimensional HSI World for Early XAI-Diagnostics of Plant Stress

**DOI:** 10.3390/e25050801

**Published:** 2023-05-15

**Authors:** Maxim Lysov, Konstantin Pukhkiy, Evgeny Vasiliev, Alexandra Getmanskaya, Vadim Turlapov

**Affiliations:** Department of Mathematical Software and Supercomputing Technologies, Lobachevsky University, 603950 Nizhny Novgorod, Russia

**Keywords:** explainable artificial intelligence, hyperspectral image, thermal IR training, zero-shot learning, plant stress, early diagnosis

## Abstract

This work is mostly devoted to the search for effective solutions to the problem of early diagnosis of plant stress (given an example of wheat and its drought stress), which would be based on explainable artificial intelligence (XAI). The main idea is to combine the benefits of two of the most popular agricultural data sources, hyperspectral images (HSI) and thermal infrared images (TIR), in a single XAI model. Our own dataset of a 25-day experiment was used, which was created via both (1) an HSI camera Specim IQ (400–1000 nm, 204, 512 × 512) and (2) a TIR camera Testo 885-2 (320 × 240, res. 0.1 °C). The HSI were a source of the *k*-dimensional high-level features of plants (*k* ≤ K, where K is the number of HSI channels) for the learning process. Such combination was implemented as a single-layer perceptron (SLP) regressor, which is the main feature of the XAI model and receives as input an HSI pixel-signature belonging to the plant mask, which then automatically through the mask receives a mark from the TIR. The correlation of HSI channels with the TIR image on the plant’s mask on the days of the experiment was studied. It was established that HSI channel 143 (820 nm) was the most correlated with TIR. The problem of training the HSI signatures of plants with their corresponding temperature value via the XAI model was solved. The *RMSE* of plant temperature prediction is 0.2–0.3 °C, which is acceptable for early diagnostics. Each HSI pixel was represented in training by a number (*k*) of channels (*k* ≤ K = 204 in our case). The number of channels used for training was minimized by a factor of 25–30, from 204 to eight or seven, while maintaining the *RMSE* value. The model is computationally efficient in training; the average training time was much less than one minute (Intel Core i3-8130U, 2.2 GHz, 4 cores, 4 GB). This XAI model can be considered a research-aimed model (R-XAI), which allows the transfer of knowledge about plants from the TIR domain to the HSI domain, with their contrasting onto only a few from hundreds of HSI channels.

## 1. Introduction

Here, we continue the topic of significance in explainable artificial intelligence (XAI) of information theory and, in particular, entropy, which started in our previous article by Lysov, et al. [1] (2022). This article investigates the introduction of entropy and max-min into the vector of statistical features for the problem of classifying plant states experiencing developing drought stress. They were introduced as traditional measures of the diversity of the states of the objects under study. This made it possible to reduce the number of features required for confident (with accuracy = 1) early drought detection to only two features, which must include entropy or max-min. Plant state was detected using Thermal IR (TIR) images and hyperspectral images (HSI) data separately. HSI data were represented by six popular vegetation indices and by three separate channels: R_630_, G_550_, and B_480_. All HSI-based indices and TIR were shown to be able to achieve such early detection of drought, but the TIR-based features proved to be the most sensitive and reliable.

Here, we searched for effective solutions to the same problem of early diagnosis of plant stress on an example of wheat and its drought stress. However, the main idea was to combine the benefits of two of the most popular agricultural data sources, HSI and TIR images, in a single XAI model. This will give the HSI additional functionality as a TIR sensor and increase the reliability of plant drought detection without actually using the TIR sensor on the UAV, as well as increase the temperature-based explainability of AI decisions.

Artificial intelligence (AI) has become the most popular approach to diagnosing plant stress in smart farming. Boldú, in a 2018 review [2], noted the widespread use of Deep Learning (DL) in agriculture. Kakogeorgiou, et al. in 2021 [3] and Wei, et al., 2022 [4], showed that DL, gradually adding certain properties of explainability to its arsenal, is becoming more and more used for plant stress and disease diagnostics. At the same time, hyperspectral images (HSI) of plants are widely used as initial data for AI diagnostics. This means that extending the functionality of HSI with thermal range would be very beneficial for both agriculture and AI.

### 1.1. HSI as Multidimensional World

A typical HSI occupies hundreds of megabytes of memory, has a complex multi-file structure, and requires special processing software. Each HSI pixel is an n-dimensional channel, or spectral, vector with an almost unique combination of hundreds of channel values. The presence of hundreds of channels in the spectral characteristic of each pixel for an HSI gives the object depicted in it hundreds of features and comes with the fundamental possibility of classifying the object by one n-dimensional pixel (signature) even without using context. Dozens of vegetation indices, such as the Normalized Difference Vegetation Index (NDVI), developed in Earth remote sensing (ERS) are also used in the diagnosis of the state of plants and fields. These indices are built on rational combinations of HSI channel values, and their number continues to grow [5,6]. When solving problems of diagnosing the state of plants, indices are usually used instead of HSI, but can also be used together with HSI channels and spectral characteristics of its pixels, sometimes even preferring the latter [7].

Since the use of HSI in the practice of remote sensing, the “Spectral angle” measure and the classification procedure based on it, called the Spectral Angle Mapper (SAM) Classification, have been used. In essence, this coincides with the Fisher criterion for determining separability in a multidimensional space (Grechuka, 2021) [8]. As a result, from the point of view of AI, hyperspectral images form their own multidimensional HSI world, in which both the curse of dimensionality and the blessing of dimensionality are necessarily present [9] (Gorban, 2018).

### 1.2. HSI and AI in Agriculture

The above properties of HSI have made the research and application of HSI constantly expanding [7,10,11,12,13], and applied problems using HSI have become a real testing ground for the development of AI methods and models. For example, Zhang et al., in a 2020 review [13], explored the application of AI for diagnosing plant stresses on HSI data over the past 3 decades. It has been shown that HSI signature data are able to provide recognition of plant diseases with acceptable accuracy, in terms of leaf, stem, ear, and crown. Examples of solutions for images both on the scale of traditional remote sensing and at distances of the order of a meter(s) accessible to UAVs and ground units are given. Publications [7,11,12] reflect the trend towards using “close-range” HSI and high resolution HSI as source data. Sometimes HSI is supplemented with thermal (Thermal IR, TIR) imaging [12], which is absent in the set of HSI sensors, but is specialized for early stress diagnostics by increasing plant temperature [12,14]. Early diagnosis is understood as one that avoids crop losses. The problem of early diagnostics of plant drought stress was also studied in [7]. It was shown that when using several popular classical ML methods, including Multi-Layer Perceptron (MLP), the accuracy of plant drought state detection based on the full spectral characteristic of plant HSI, as well as its derivative, turned out to be higher than the accuracy obtained on a combination of nine vegetation indices. Review [15] is devoted to the development of algorithms for detecting the quality of wheat grain protein and its relationship with nitrogen status, based on ML and HSI data, over the past almost 25 years.

In Schmitter et al. (2017) [10] proposed an original “unsupervised domain adaptation” approach for early detection of plant-drought stress, which also uses HSI data. The approach provides the transfer of a neuromodel trained for early detection of plant-1 stress to the detection of plant-2 stress, using wheat and corn as an example. The transfer is carried out on the first principles of plant biology and does not require supervision. The other original method, which uses HSI possibilities, was proposed by Pan et al. (2021) [16]. It is the “zero-shot learning” approach based on using a high-level feature vector. Such a vector ensures the minimization of a dataset required for training, as well as the transfer of training results from one dataset to another, in which some of the categories are missing (or not visible). The high-dimensional spectral characteristics of HSI are considered the source of high-level features.

### 1.3. Notions of XAI and Explainability in AI

**Figure 1:**
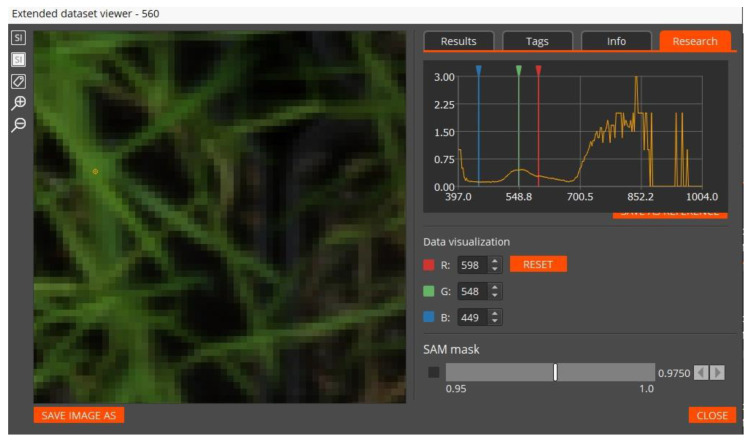
An example of hyperspectrum (**top right**) viewing for a plant pixel taken with an HSI Specim IQ camera. The pixel is marked with a light dot in the (**upper left**) quarter of the image. The plant belongs to the control group.

The number of publications devoted to explainability and explainable artificial intelligence (XAI) has increased significantly in recent years, but there is a noticeable difference in the interpretation of the content of XAI. For example, Zhang et al., which presented in their review (in [17], Figure 1) the point of view of XAI applicating in diagnostics and surgery, proposed the following scheme for covering the areas of AI, ML, DL, and XAI: ML entirely belongs to AI, and DL belongs to ML; XAI is the part of AI that overlaps with ML but does not overlap with DL. This vision is consistent with previous publications [18,19,20]. In Lundberg et al. (2018) [19] build explainable prediction and prevention models for hypoxaemia during surgery on objective patient monitoring data, substantiating their importance with the data of Shapley value analysis. In Lamy, et al. (2019) [18] explain the prediction for breast cancer using a case-based reasoning approach, which is typical for the medicine. In Antoniadi et al. (2021) [20] draw attention to the lack of explainability in ML-based Clinical Decision Support Systems (CDSS) for the clinicians operating them and insist on its increase. It is understood that for other applications of XAI, “linician” means a person who directly uses XAI predictions.

Some works explore XAI as a universal tool [21,22,23,24]. The authors of Wang et al. (2019) [21] proposed an original “Theory-Driven User-Centric” approach for XAI designing. According to them, “explainability” should be oriented towards the user, making it “User-Centric” using the methods of cognitive psychology and the laws of human thinking. The main idea of the approach is expressed by the formula “Understanding Humans Should Inform Explaining AI”.

Vilone and Longo (2020) lead a systematic 81-page review of XAI based on 361 sources [22]. The review contains a detailed study of XAI, including a detailed analysis of concepts related to the concept of explainability, a discussion of explainability attributes, a classification of existing methods, and approaches to designing XAI elements and systems. They generalized a state-of-the-art hierarchical framework for XAI design, which includes the following components (from bottom to top): data; modeling; XAI methods; explanators; and evaluation. The authors also proposed an alternative ideal vision of XAI as a system of nested components. The central and main focus component is the Explanators (textual, pictoral, rules, dialogue, mix-formats), which is what end-users will ultimately interact with. Then follows Attributes (comprehensibility, interestingness, persuasiveness…), Modeling (AI connectionist learning, AI symbolic reasoning), and Evaluation of explainability (which is realized via interface interaction and human-in-the-loop technic, for designer and end-user). This Explanators-driven XAI concept appeared very interesting on the condition of considering Explanators paired with Data.

Averkin (2021), in the review [23], explores, against the background of modern publications, the possibilities and prospects for the development of XAI in the project “DARPA’s explainable artificial intelligence (XAI) program”, presented in the report [25]. This project attracted the attention of the author with its focus on deriving an explainable model from any black box model. For this goal, it was planned to create modified DL methods that study explainable features, and methods that explore more structured, interpretable causal patterns, as well as model induction methods. Maybe it means a reasonable shift of the DL model properties to the side of XAI model properties. The list of modern publications by the author also includes publications of two standards [24,26] on XAI.

The draft NISTIR 8312 (2020) for XAI definition [24] from the National Institute of Standards and Technology (NIST) is more interesting because it offers “Four Principles of Explainable Artificial Intelligence” underlying XAI with an emphasis on human-computer interaction. These principles are: (1) Explanation; (2) Meaningful; (3) Accuracy of explanation; (4) Limits of knowledge (the system must note any cases for which it was not designed). The principles are independent from the AI area, DL and classical ML, but subject both areas to the same criteria, including the 4th, which is very hard to execute for DL. Their short explanation:

Explanation: Systems deliver accompanying evidence or reason(s) for all outputs.
Meaningful: Systems provide explanations that are understandable to individual users.
Explanation Accuracy: The explanation correctly reflects the system’s process for generating the output.
Knowledge Limits: The system only operates under conditions for which it was designed or when the system reaches sufficient confidence in its output.

In the examples of providing explainability in the practice of diagnosing plant stress based on DL [3,4], we can speak so far only of partial implementation of three principles, (1) Explanation, (2) Meaningful, and (3) Accuracy of explanation, in order to overcome some of the limitations of the black-box nature of DL models. In examples of providing explainability based on ML methods [1,7,14], one can see the implementation of the same 3 XAI principles, but with a high degree of content, as well as a part of the content of the 4th principle, which has not yet begun to be given due attention in practice.

According to Linardatos et al. (2021) [27], the popularity of the search term “Explainable AI” (XAI) over the years, according to Google Trends, peaked in 2020. The XAI is considered here as a source of “white-box” models, which are the alternative to “black-box” ones. At the same time, XAI is increasingly expected to include the transfer of knowledge from one area to another as one of its main functions, also expected of such white-box models, which will be able to perform well not only in a single task.

This subsection directs us to search for an XAI solution in the form of a “white-box” model that provides the transfer of knowledge from one known area, which is changing the plant leaf temperature during the drought, registered by TIR sensor, to another area, which is the multidimensional HSI world. Additionally, our version of the white-box model was planned to be constructed according to the ideal explanators-driven XAI vision of authors [22], as a system of nested components, and under the constructive vision of the four principles of [24,26].

### 1.4. Theory and Practice of Feature-Space Dimensionality Reduction in AI-HSI Applications

The second goal of this work is to reduce the dimension of the high-level feature vector needed to train HSI signatures on the TIR temperatures, as much as possible. The practical possibility of lowering the HSI dimension in the early diagnosis of plant stress was shown by Agilandeeswari et al. (2022) [28]. They managed to reduce the number of HSI channels used for the task by about five times, from 224 to 42–45, using the selection of channels according to efficiency criteria. The criteria used for this goal were justified by the authors as dictated by the frequency of their application in the respective wavelength ranges. So, the criteria for the three ranges are: the visible (VIS) range—the value of the entropy (informativity) of the current channel; the near infrared (NIR)—the value of the Normalized Difference Vegetation Index (NDVI); the shortwave IR (SWIR)—the value of the Modified Normalized Difference Water Index (MNDWI). They selected 14–15 channels from each range. In our case the SWIR range is absent.

There are also theoretical and practical reasons for even more significant dimensionality reduction. For example, Allegra et al. (2020) in [29] confirmed that a small number of variables is often sufficient to locally describe high-dimensional data. This minimal number of variables is called the intrinsic dimension of the data (IDD). Additionally, such IDD can be viewed as a simple topological feature that is sufficient to implement unsupervised segmentation of multidimensional data.

Even more interesting possibilities are opened by Albergante et al. (2020) [30], confirming the possibility of modeling the internal structure and geometry of a complex multidimensional dataset in the form of an area graph with a low local internal dimension. The ElPiGraph software has now become part of the Python Package for Intrinsic Dimension Estimation, called the “Scikit-Dimension” [31]. Authors, Gorban et al. (2021) [32], solve the problem of constructing simple correctors that can update the properties of the AI system. However, it is essential for our study that the authors accept as a working hypothesis that the data can have a rich fine-grained structure with many clusters and corresponding peaks in the probability density. Additionally, this hypothesis, workable for the authors, confirms for us the possibility of a sharp decrease in the dimension of the space inside the grain and the efficiency of using local metrics. As an effective tool for coordinating a local subject area, it is proposed to use methods of principal component analysis (PCA).

The considered material gives us fundamental confidence that the local problem of temperature coordination on the pixels of the plant mask is solvable in the space of channels on a relatively small number of channels. However, we are forced to abandon the use of PCA, as our goal is to achieve greater generality in our solution, and PCA is context-sensitive. We find an important confirmation of our choice in a recent publication, Wei K. et al. (2022) [4], which studied a number of different datasets of fruit leaf images and demonstrated that the convolutional block attention module (CBAM) can significantly improve the feature extraction ability in applying a deep convolutional neural network model. However, the main result for us is that the attention objects and feature extraction results of the model in different agricultural classification tasks turned out to be different.

## 2. Materials and Methods

### 2.1. Materials

An experiment on the early detection of drought stress in plants was carried out in the biolab of our university. Drought studies were carried out on wheat plants (*Triticum aestivum* L.), cultivar Zlata. After soaking, the seeds were planted in plastic pots 10 × 10 × 8 (sm) filled with universal soil “Morris Green”, 16 plants per pot. The temperature was kept at 24 °C and the air humidity was about 50%. Plants were illuminated with luminescent lamps with intensity about 200 µmol/s/m^2^ with a 16-h photoperiod. Plants were grown up to the age of 14 days, after which they were divided into control and experimental groups. In the experimental group, watering was stopped. In the control group, it was continued according to the standard schedule (1 time in two days). For ease of observation, plant pots were placed in 3 boxes of 30 pots each. The control group has 15 pots in the left half and the experimental group has 15 pots in the right half of the box.

During 25 days the state of the plants was regularly recorded, every 2–3 days from a height of about 1 m and at a right angle, with the following cameras: (1) Specim IQ hyperspectral (HSI) camera (range: 400–1000 nm; spectral resolution: 3 nm; channels: 204; 512 × 512 pix); (2) Testo 885-2 thermal infrared (TIR) camera (320 × 240 pix, temperature resolution is 0.1 °C). The spatial resolution of the HSI camera was 1.5 mm per pixel, and for the TIR camera was 2.4 mm per pixel. TIR sensors were chosen to directly record the leaf temperature, an increase in which is the earliest feature of a stress condition, see Jackson et al. (1981) [33]. Images with HSI and TIR sensors were taken in the morning 2–3 h after the illumination was turned on. An example of an RGB interface for selecting a plant or soil pixel on the HSI and visualizing its recorded spectrum can be seen in Figure 1. The images were recorded for the following 11 days of the experiment: 1st, 3rd, 6th, 8th, 10th, 12th, 14th, 16th, 19th, 22th, and 25th. The total image volume was about 72 GB, mainly comprising HSIs.

**Figure 2:**
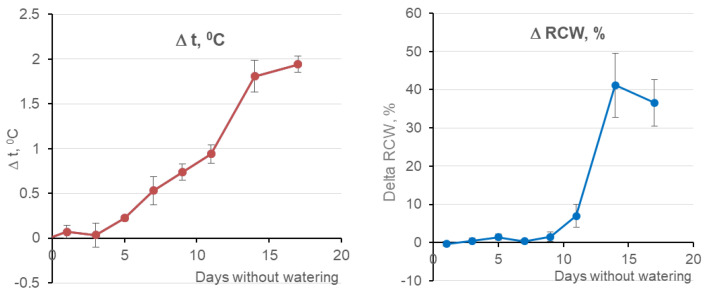
The differences (Δ) between the plants in the control and experimental groups: in average leaf temperature, according to TIR images (**left**); in average relative water content (RWC) in wheat leaves (**right**). Day 0 is the last day of watering for the experimental group of plants.

In parallel with image recording, objective measurements of differences between the plants in the control and experimental groups in leaf temperature (according to TIR images, °C) and water loss (%) were performed (Figure 2).

The water loss measurements were carried out under standard conditions of temperature and humidity. The relative water content (RWC) in wheat leaves was estimated from the ratio of fresh weight (FW) and dry weight (DW) of the leaves. The plant leaves were dried for 48 h at 70 °C to obtain a dry weight value. The RWC was calculated by the expression: RWC = (FW − DW)/FW. Plants for measuring water loss were selected from the experimental and control groups, grown according to the same scheme, but without recording optical data.

The following key events and changes in the state of experimental plants were detected (Figure 2): (1) an increase in the average temperature of plants by 0.2 degrees on the 5th day without watering; (2) the beginning of water loss by the plant after 11 days (on average about 8% of the volume of water). The first event is the earliest feature of the onset of drought stress and occurs for the plant without the losses of water and green mass. Such detection of a plant stress event is a criterion of “early” detection success. Outside the range of Figure 2, on the TIR images of the segment 17–25 days, we also have another key day. It is on the 19th day that we have a fracture in the line of a monotonous increase in plant temperature, which may be due to the depletion of the compensatory function of wheat plants. The days {1,3,6,8,12,19,25}, in which notable changes in plant temperature or water loss can occur or are occurring were selected as 7 key days.

To execute the study, we organized 2 datasets for training and testing purposes by dividing the experimental part of box #3 of the dataset into 2 parts named “Train” and “Test” (Table 1). This ensured comparability of parts of this study. Plant pots were selected for the best focus TIR-image criterion.

### 2.2. Construction of XAI Early Diagnostics Network on the Input Explanators Having Only High-Dimentional I Data as the Source

Our main goal and problem here is to combine the benefits of two of the most popular agricultural data sources: hyperspectral images (HSIs) and thermal infrared images (TIR) to provide the agriculture plants health analysis. According to the above publications [9,16,22,27,34], the best guiding idea for building an XAI neural network for this goal is an explainable user-centered “white-box” model, which has explainable input, explainable output, and provides “the transfer of knowledge from one area to another”. In our case, the first area is TIR images, and the second one is the HSIs. Both areas have the same users: agronomists and biologists, and both are explainable for them.

The HSI is initially unaware of the plants’ temperature, but its spectral characteristics can be used as a source of high-level features. According to [16] the presence of high-level features is a necessary condition for training to “unseen categories”, but this presence should be confirmed during our study, starting from the full dimension of the spectral characteristic. So, our XAI neural network should ensure the transfer of knowledge about the plant’s temperature to HSI signatures, if it is possible. After this it is important to localize the most important channels, which are the real high-level features, and minimize their number.

Then as the main study result, we could consider our trained XAI neural network as a Research XAI (R-XAI) block, which ensures the transfer of the temperature knowledge onto to HSI signatures (which was impossible before) due to knowledge of the most important channels for this. Such an RXAI unit could be widely used, including for early stress diagnostics not only in wheat plants, considering the HSI sensor to be sufficient for all tasks.

**Figure 3:**
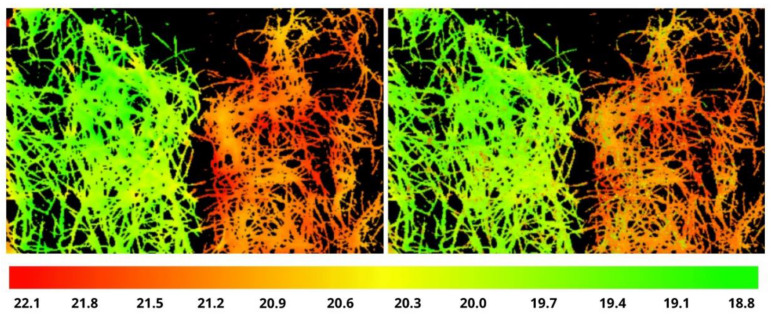
The original image from TIR sensor in color scale (**left**), the result of the temperature prediction (**right**) (courtesy of the authors of [34]).

The confidence in solving the problem is based on a number of fartors. The first one is our initial experience of training hyperspectral images (HSIs) to the TIR temperatures [34] based on a “Zero-Shot Learning” approach [16]. Unfortunately, (1) the achieved *RMSE* value (0.52 °C, 5 times the TIR resolution) is obviously not enough for practice, and (2) the reconstructed temperature range [18.8–22.1 °C] had a significant gap of 1 °C in the middle due to the same gap in the used original data (1st box at 25th day, having the widest temperature range, see Figure 3).

Another group of factors are long-term studies aimed at finding objective biophysical relationships between the state of plants with both the shape of their reflected hyperspectrum in general and individual channels in particular. This is a series of works since 1970 [35] (Knipling, 1970), where the effect of chlorophyll and water in a wide range of the hyperspectrum from 400 to 2800 nm has been explained. The most frequent result of these works was the proposal or clarification of HSI-based vegetation indices, as in [36] (Gitelson, 1996), where a new vegetation index R750/R700 for the “red edge” zone of hyperspectrum was proposed, and the linear relationship between the chlorophyll concentration and the index value was established. Similar studies continue to the present [37]. These studies show that there are both HSI ranges and individual channels that are most sensitive to the state of plants.

### 2.3. Building a Plant Mask for Automation of HSI Markup and Tools for Correlation and Regression Research Based on It

To solve the problem of training HSI signatures from TIR temperatures, it is necessary to mark up the HSI pixels with the TIR sensor temperatures for all 7 key days. Moreover, this must be completed for such a number of pots with plants, which will provide the number of pixels of plants sufficient for training. To automate the markup process, 512 × 512 HSIs were aligned with 320 × 240 TIR images using the homography transformation. Combining images of significantly different resolutions created additional difficulties: (1) to improve accuracy, alignment was performed at the level of each pot with plants instead of boxes; (2) along the border of the plant leaf, large pixels of the TIR image appeared, which overlapped both the leaf and the soil, having different temperatures.

To study the correlation of the TIR pixel temperatures with the pixels of each HSI channel, as well as to perform training on pixels that belong to plants strictly completely or only partially, it is necessary to provide a tool for separating pixels that have different levels of plant and soil mixing. It should be a tool to create and manage a plants’ mask (*M_j_*, where *j* is an ordinal number of key day from 1 to 7).

**Figure 4:**
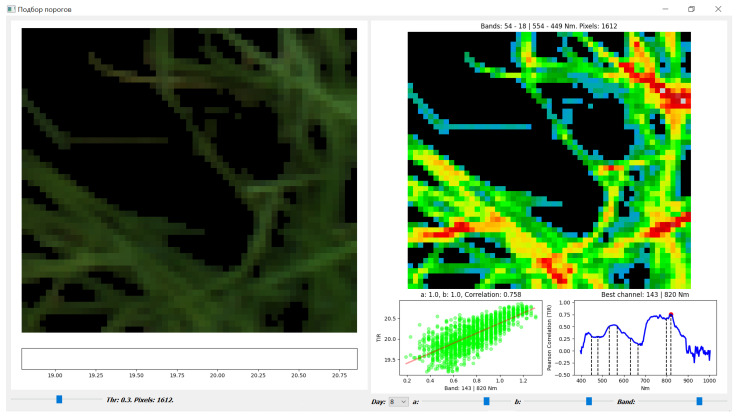
Interface for building and studying plants’ mask (for HSI and TIR), cloud and regression, equipped with control of *Band*, *Thr*, *a*, *b* parameters, and correlation and regression plots.

To solve this problem, the following steps were taken:

(1) A plants’ mask managed by a threshold (*thr*), which separates plant from soil, was constructed. For this goal the vegetation index *NDblue* (1) [1] was used. The condition of belonging to the plants’ mask is *NDblue* ≥ *thr*.
(2) A special tool with interface (Figure 4) for the plants’ masks setting has been implemented. This tool removes from the mask those points of the point cloud (PC) that have deviated from the linear regression by more than *a* up or *b* down. This can provide both homoscedasticity of the regression and control over the size of the standard error of the regression. Parameters *a* and *b* can vary independently, but the study used only the symmetrical variant *a = b*.

(1) $$NDblue=\frac{V}{\left(max\left(V\right)-min\left(V\right)\right)},\;V=R_{550\;}-R_{450},\;NDblue\;\geq \;thr$$

Figure 4 shows plants in one of the pots (left) stained with three HSI channels: R_680_, G_550,_ and B_480_. Under this image the current scale of TIR values is shown. The threshold (*thr*) is connected with the amount of soil allowed in a pixel classified as a plant. *thr* values from 0.1 to 0.5 (lower left slider) change the allowed proportion of soil from exceeding the plant presence to zero (correspondence was established visually). Near *thr* value for the slider, the value of mask *M_j_* area (Pixels) is shown.

On the right, we can see a pseudo-color TIR image for the pixels under the current plant’s mask *M_j_*. Below this image:

Right plot: Correlation plot between TIR and all HSI channels for the day of the experiment (Day field in the bottom panel); the channel with the best correlation is shown in text form above the plot. To assess the correlation, the Pearson Linear Correlation Coefficient (PLCC) is used according to Formula (2). Using the Band slider below the graph, you can set any channel (usually the best in correlation) for which you want to generate a point cloud. The selected channel is highlighted on the chart with a red mark.

Left plot: Point cloud plot (X-axis—channel HSI values; Y-axis—TIR values) for building a linear regression of the temperature prediction. Below it are sliders for setting parameters *a* and *b*. The initial values *a = b =* 1 do not limit the cloud and mask. Reducing *a*,*b* to 0.5 has a minimal effect (reducing the cloud/mask by 2% or less), to 0.1—reduces the mask by 2–2.5 times, providing regression homoscedasticity and reducing its *RMSE*.

(2) $$r(x,y)=\mathrm{cov}(x,y)/(\sigma x\cdot \sigma y)=E[(x\;-\;Ex)(y\;-\;Ey)]/\sqrt{Dx}\cdot \sqrt{Dy}.$$

The actual values of the parameters for the images and graphs presented in Figure 4 are as follows: *Day* = 8th; HSI channel or *Band* = 143/820 nm; *thr* = 0.3; *a = b =* 1. Channel numbering is used starting from 0.

With the help of such a tool, we can explore building both a plants’ mask and the linear regression on the following parameters: *day*; *HSI channel* (or *band*); *thr*; *a*, and *b* to detect the presence of a high correlation of any from *HSI channel*(s) with TIR image andbuild a predictor *y*(*x*) for TIR-HSI cloud in the form (3).

(3) $$y_{i}=Ey+\frac{\sigma_{y}}{\sigma_{x}}(x_{i}-Ex),$$

where *x_i_*—*i*-th mask-pixel value for current HSI channel, *y_i_*—*i*-th mask-pixel value for TIR. 

### 2.4. Dimensionality Reduction and Building of the XAI System Based on HSI-Input-Data as the Source of High-Level Features

Following the main ideas of [32], we also plan to use the experience of biologists and agronomists in the search for channels that are sensitive to plant health.

We plan to solve the problem of mixing the benefits of HSI and TIR based on the HSI input data in two ways: (1) to investigate whether among the HSI channels there is one or the other that has a high correlation with TIR, close to a functional relationship; (2) training HSI pixels with TIR values using the XAI model and searching for high-level features [16] (H-LF). The second goal is to try to minimize the number of HSI channels required for the solution. Probably the most important thing is to find a solution for the 2nd problem, which will depend not on the key day, but only on the stress state level of the plants, and to achieve this on the reduced number of HSI channels, which will be H-LFs. This means that the XAI structure must provide for the possibility of changing the number of HSI channels (*k*).

**Figure 5:**
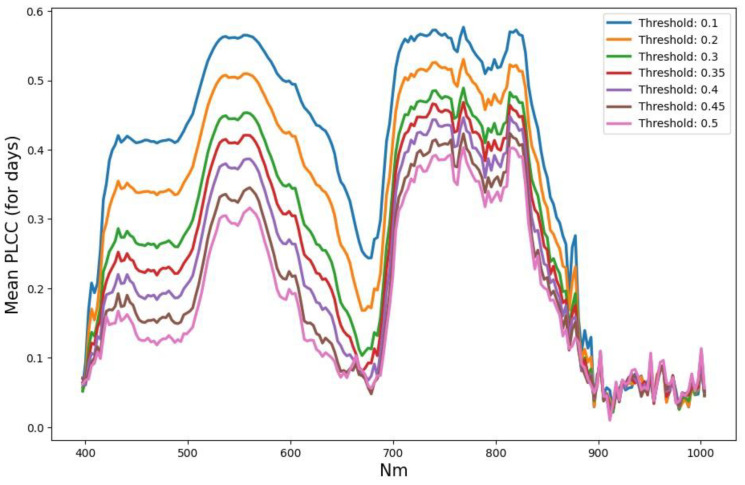
PLCC absolute values averaged over key days for various threshold values driving the mask, without any restrictions by *a*,*b* parameters (*a = b =* 1). The x-axis is the wavelength for HSI channels (nm).

Let us analyze the absolute PLCC values averaged over key days for various threshold values (Figure 5) to help us in reducing the number of HSI channels. The PLCC values were calculated both as the average over key days (as in Figure 5), as well as separately for each key day. This was completed for both the ‘Train’ and the ‘Test’ parts to compare the correlation patterns of these parts.

It can be seen that the correlation curves for different thresholds have very close shapes, which can be characterized by several extreme points near the following (channel number/wavelength): 59/570, 97/680, 117/740, 126/770, and 143/820 (4 max + 1 min), the first 3 of which are included in the expressions of popular vegetation indices. Some widely used vegetation indices (VI), such as NDVI (Normalized Difference Vegetation Index), GI (Greenness Index), PRI (Photochemical Reflectance Index), and others [10], can also be included as additional features. Together, it will be only *k* = CHextr + VIs channels/indices (hereinafter referred to as channels) instead of all 204. The real number of local maxima is much more than 5, and they change from one key day to another. How many highs/extremes will be enough for each day and for all days together, and what their types will be, should be a subject of study.

**Figure 6:**
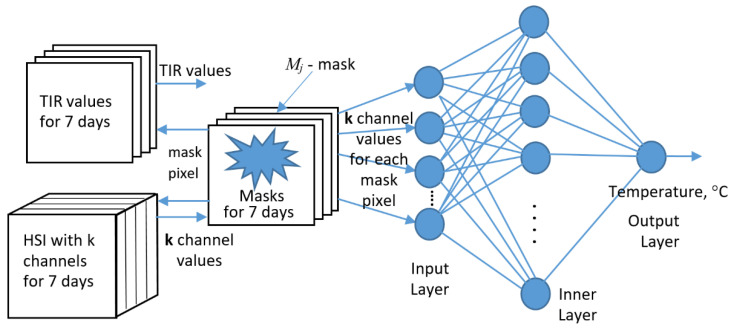
The structure of the XAI neural network implementation and its SLP regressor.

So, to build the XAI temperature regressor, we use 3 data sources at its input for each of the 7 key days: the values of HSI(j), TIR(j) and *M*(*j*), where j is the ordinal number of the key day (Figure 6). We will use SLP (Single Layer Peceptron) as the base ML structure for XAI. As an input HSI feature vector for predicting the TIR value, we will use pre-selected *k* HSI channels, which should ensure the prediction of the temperature value with acceptable accuracy. The problem of choosing *k* and its minimization will be solved in the course of the study.

We will use the Mask component as a sampler of HSI and TIR pixels to build a cloud and parameterize our study in terms of {*thr*, *a*, *b*} (see Figure 4). It is important to note that the masks for each key day are different.

As a loss function, we use *RMSE* as an estimate of the deviation of the temperature predicted for an HSI pixel from its value in TIR for each of the key days. In this case, the loss function should take the following form (4):

(4) $$RMSE=\sqrt{\sum_{j=1}^{7}\sum_{m\in M_{j}}{\left(py_{jm}-y_{jm}\right)}^{2}/n},$$

where *py_jm_*—temperature predicted from the *k* channels of the *m*-th HSI pixel of the *j*-th day mask *Mj*, $y_{jm}$—*m*-th pixel value of TIR mask *Mj*, *n*—total number of pixels in 7 masks.

Three blocks at the input of the XAI neural network (Figure 6) implement automatic markup with temperatures and the formation of a (*k* + 2)-dimensional cloud at the input of the SLP regressor, where *k* is the number of HSI-channels used.

The remaining 2 measurements are the temperature and the day of the experiment. In the case of *k* = 1 and one day, we get a two-dimensional cloud, as shown in Figure 4. The HSI block provides a selection of a given number of given or arbitrary channels.

Key day sampling management provides both the training on the cloud for all days, and for each day separately. The previously shown mask and cloud control parameters also work: *thr* and *a*,*b*.

## 3. Results and Their Discussion

### 3.1. HSI-Input-Data Dimensionality Reduction via Searching Channels That Can Be Consided as High-Level Features

According to the order planned in the Section 2.4, at first we solved the task (1) to investigate whether among the HSI channels there is one or the other that has a high correlation with TIR, which is close to a functional relationship.

**Figure A1:**
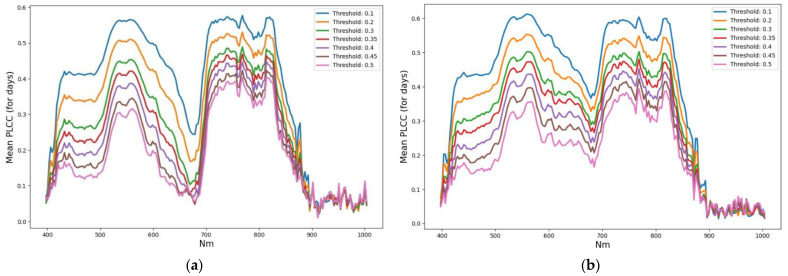
Comparison of the mean over key days PLCC plots for TIR with all HSI channels for 2 datasets of the experiment: (**a**) “Train” and (**b**) “Test”.

We have PLCC absolute values, which were calculated for both the ‘Train’ and the ‘Test’ parts to compare the correlation patterns of these parts. It turned out that plots averaged over key days for the ‘Train’ part and the ‘Test’ part have noticeable differences. This is expressed in a noticeable rise of ‘Test’ plots for the zone of minimum (channels 96–97, 680 nm), and, with it, for the entire left part of the plot (see Figure A1, Appendix A). On this basis, it can be assumed that the part of the spectrum to the right of 740 nm is more promising for the search for high-level features. The highest was the average PLCC for the 143 channel (820 nm).

The nature of the behavior of the plant mask (Mask), correlation coefficient (PLCC), channel (Best Ch.), the best in PLCC, and the *RMSE* value when changing the Threshold and *a*,*b* parameters is illustrated in Table 2.

Based on Table 2 together with the study interface (Figure 4), the following conclusions can be drawn. As *a* and *b* decrease, the correlation curve changes and the maximum correlation channel shifts towards the 143/820 nm channel. The *RMSE* value for channel 143 is always significantly lower than for other channels. A study of the correlation of TIR values with vegetation indices showed twice and lower PLCC values than for the channels.

Studies of the possibilities of temperature prediction by Formula (3) based on the 143rd HSI channel (820 nm) with an analysis of the behavior of the Mask, PLCC, and *RMSE* values in the parameters {key *Day*, *thr*, *a*,*b*} are presented in 3 tables: Table A1, Table A2 and Table 3.

Table A1 analyzes, on the example of the Train dataset, the behavior of the Mask, PLCC, and *RMSE* in the full range of *thr* = 0.1–0.5 with constant initial values *a = b =* 1, and also demonstrates statistics of both the plant’s temperature in TIR values and 143 channel values.

Table A2 analyzes the situation when we decrease the initial values *a*,*b* ten times or more, and compared with PLCC and *RMSE* values inside a Mask ‘in’ truncated by reduced values of *a* and *b*, and *RMSE* for a mask ‘out’ of the Mask ‘in’, but in the full Mask (Table A1). The best values are highlighted with a light gray background. The table shows that ‘Out’ *RMSE* values are 1.5 to 4 times larger than ‘in’ and on 0.2–0.7 °C larger than *RMSE* for the full Mask.

Table 3 is an extraction from Table A2 for *thr* = 0.4 and 0.5 only without columns of ‘TIR in’ statistics for a compact presentation of the results.

It was found that:

(1) The leaders in correlation with TIR in terms of the average PLCC when *thr* changes are the channels of the NIR range (112–144 channels), followed by visible green channels. Channel 143 was identified as the leader.
(2) During variations of the *thr* value from 0.1 to 0.5, the area of the plant mask decreases approximately linearly, by a factor of two–four depending on the key day. The area of the plants’ mask reaches a maximum of 2823 pix (near 7/8 of the image) and a minimum of the variation (less than two times) on day 12.
(3) Each step increasing the threshold (*thr*) actually removes pixels with reduced soil content from the original mask. The value *thr* = 0.45 eliminates almost all mixed pixels, and *thr* = 0.5 guarantees that the pixel belongs to the inner part of the sheet.
(4) The PLCC value on each day is maximum at *thr* = 0.1 and decreases monotonically by 1.5–3 times with an increase in *thr*, and by day the PLCC maximum is on the first day of the experiment (0.75 for channel 143).
(5) According to Table A2 and Table 3, the usefulness of reducing the parameters *a*,*b* at high threshold values (*thr* = 0.4 or 0.5) is sharply reduced, as it leads to mask depletion. As a result, the accuracy of the forecast increases, and outside the truncated mask it even deteriorates slightly. For these thresholds, values of *a*,*b* closer to 0.5 should be recommended.

### 3.2. The Problem of Training HSI by Plant’s Temperatures from TIR-Values Using the Built XAI Neural Network and Reducing the Dimension of the Feature Space

The solution of the problem via built XAI network is possible in two ways: (1) as a set of individual solutions for each of the key days; (2) as a single solution that depends not on the day, but on the objective state or stress level of plant leaves, recorded by the HSI and TIR cameras.

Solution 1 is equivalent to assigning an additional feature, such as a number of the key day. In this case, we must use the test dataset with the same feature.

Solution 2 is more interesting, as it should practically expand the range of the HSI sensor with the capabilities of the TIR sensor in a part of the plant study. We explored both solutions to compare and better use their capabilities.

Solution 1. Channel 143, as the most correlated with TIR, was tested first as a high-level feature. To combine training for separate key days into one process, the number of the key day was added as the feature. The result of studying the influence of the parameters {Threshold; *a*,*b*} on both the temperature prediction accuracy in *RMSE* values and the Mask area for Train and Test datasets is shown in Table 4. The best results are highlighted by the background.

From Table 4 we can see the following:

(1) All best results are near the TIR resolution of 0.1 °C and took place at *a = b =* 0.1 regardless of *thr* value. The worst *RMSE* values are near 0.2 °C at *a*,*b* equals 1 and 0.5.
(2) The *RMSE* value is highly dependent on *a*, *b* and hence the area of the mask. Reducing the mask by about half improves the *RMSE* value by about two times.

However, when the mask is reduced by reducing *a*,*b*, the *RMSE* calculation is performed on a part of the original cloud that is closer to its linear regression. In summary, *RMSE* = 0.2 °C appears to be more reliable. Additionally, we can conclude that the temperature prediction in the local coordinate system 2D {channel-143 value; key day} provides an *RMSE* value of around 0.2 °C.

We have used “day” here as a simple second feature to validate the feasibility of building a 2D local feature system implemented in our Research XAI-neural network. The choice of a practically useful and reliable second coordinate requires additional research.

Solution 2. This solution is aimed at finding the minimum set of HSI channels capable of forming a high-level feature vector for training plant HSI pixels on the temperature values of the TIR sensor.

To do this, a number of solutions to the learning problem were constructed on a successively narrowing set of channels: 204 channels (full spectrum); 146 channels, from 15 to 160 (the least noisy part of the spectrum in the experiment); 32 channels (selection of correlation extrema with TIR); 8 channels (combination of channels used in indices and PLCC extrema); and 7 channels (removal of 95 channel from 8).

The temperature prediction accuracies achieved for the indicated series of channel samples are presented in Table 5.

Table 5 shows the results of training on five sets of his channels, including, respectively: 204, 146, 32, 8, and 7 channels. For 204 channels, the whole nature of the dependence on the parameters *thr* and *a*,*b* is shown. It can be seen that for *a*,*b* = 0.1, the value of Train *RMSE* increases for Test *RMSE* by about two times (in the lower row of 204 series, by three times). The ratio of Train and Test is more stable for *a*,*b* = 0.5. Therefore, in predicting the real accuracy, one should rely more on the data for *a*,*b* = 0.5.

For the remaining four variants, the nature and magnitude of the dependence on *thr* remain very close to the first one. Therefore, only the last two lines of similar dependencies are shown. The values for the Test *RMSE* column with parameters {*thr* = 0.5; *a*,*b* = 0.5} are, respectively: 0.20; 0.24; 0.27; 0.27 (°C). This suggests that the found combinations of 32, 8, and 7 channels are almost equivalent to the full set of 204 channels, and the found 7 channels are a necessary high-level features or very close to them. A further decrease in the number of channels is impractical without automating the procedure for finding their best combination.

**Figure 7:**
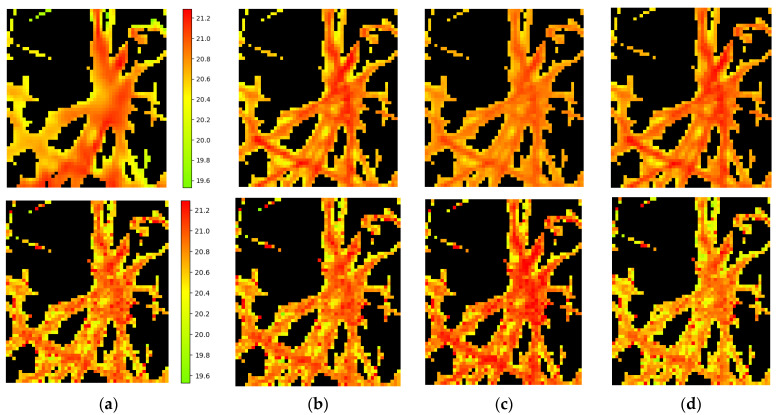
From **left** to **right**, in the same color scale, the images for one pot on key day 12. ***Row 1***—(**a**) TIR image; the approximation by Formula (3) for cases Table 3: (**b**) *thr* = 0.4; *a*,*b* = 1; *RMSE* = 0.1; (**c**) *thr* = 0.4; *a*,*b* = 0.1; and *RMSE* = 0.3; (**d**) *thr* = 0.5; *a*,*b* = 1; and *RMSE* = 0.21. ***Row 2*** (for Table 5, 8 **, 7 **)—(**a**) 8 **; *thr* = 0.4; *a*,*b* = 0.5; and *RMSE* = 0.29; (**b**) 8 **; *thr* = 0.5; *a*,*b* = 0.5; and *RMSE* = 0.27; (**c**) 7 **; *thr* = 0.4; *a*,*b* = 0.5; and *RMSE* = 0.29; (**d**) 7 **; *thr* = 0.5; *a*,*b* = 0.5; and *RMSE* = 0.27.

A visual series that deepens the understanding of the obtained results is shown in Figure 7. 

Here we can see the following:

Row 1—(a) the linear interpolated TIR image; approximation by Formula (3) for cases Table 3: (b) *thr* = 0.4; *a*,*b* = 1; *RMSE* = 0.1; (c) *thr* = 0.4; *a*,*b* = 0.1; and ‘*RMSE* out’ = 0.3; (d) *thr* = 0.5; *a*,*b* = 1; and *RMSE* = 0.21; and *RMSE* = 0.1 °C; (4) approximation by Formula (3) with *a*,*b* = 0.1 and also *RMSE* = 0.1 °C, but for ‘Mask in’, which is truncated to 794 pix from the full Mask = 1954 pix (see Table 3, Table A1 and Table A2). The real value of ‘*RMSE* out’ in this case equals 0.3 °C).
Row 2 (for Table 5, 8 **, 7 ** which have very close *RMSE*)—(a)) the result of training our R-XAI block on *k* channels using the plants’ masks with parameters *k* = 8, *thr* = 0.4, *a*,*b* = 0.5; (b) the same for *thr* = 0.5; (c,d) are the same as previes two but for parameter *k* = 7. It can be seen that results for 7 ** and 8 ** are more noisy than in Row 1. This may be due to the use of data from different key days in the one common training process.

## 4. Conclusions

An attempt was made to develop the XAI apparatus on the example of an actual applied problem of early XAI diagnostics of plant stress using only hyperspectral image (HSI) data. The choice of HSIs as a data source is due to the fact that their pixels are represented by hundreds of high-resolution channels (units of the nm), and this is widely used today in general diagnostics of plant health. The aim of the study was to provide a level of explainability in the early diagnosis of plant stress for HSI data equal to the level for data from the TIR sensor (320 × 240, 0.1 °C), which is able to sense an increase in plant temperature (about 0.2 °C, see Figure 2) caused by a change in transpiration (evaporation of water by the plant) at the earliest stage of the stress. 

This goal was achieved by training the HSI pixels of plants by the temperature value from the TIR pixels via the XAI model and the zero-shot learning approach for Unseen Categories [16]. This model training is possible on an arbitrary sample of HSI channels selected according to some criterion. The training problem was solved as a problem of transferring knowledge about plants from a TIR sensor to any number (*k*) channels of hyperspectral images (HSIs) of plants, which would be able to predict the temperature with a given *RMSE* value.

Choosing the minimum of *k* is a research (R) problem based on the XAI model, which can be considered as a special block of R-XAI. Such an experimental block R-XAI, focused on the early diagnosis of plant stress, was implemented and used in the course of this study.

The structure of our XAI model is built on the inclusion of an SLP regressor with an HSI automatic marking up block at the input. It provides an HSI temperature prediction with an *RMSE* error of 0.2–0.3 °C, which is acceptable for early diagnostics. Using the R-XAI block, the dimension of the model, equal to the number of HSI channels carrying knowledge about TIR, is reduced from 204 to several (seven–eight) channels (which is five–six times better than in [28]). The transferring knowledge of temperatures to HSI channels also includes determining the list of knowledge-bearing channels.

As a result, our special XAI block can be considered a research-aimed one (R-XAI), which allows the transfer of knowledge about plants from the TIR domain to the HSI domain, with their contrasting onto only a few from hundreds of HSI channels. 

Particular solutions have been obtained that provide *RMSE* within 0.2 °C. The model is computationally efficient in training; the average training time is much less than 1 min (Intel Core i3-8130U, 2.2 GHz, 4 cores, 4 GB).

During the study for correlation of each of the HSI channels with the TIR image, the existence of a channel in the NIR range, demonstrating a high correlation with TIR, has been established. This is the 820 nm channel, on the basis of data on which the linear temperature approximators can be built. The channel is not yet used in the construction of popular vegetation indices.

A step-by-step decrease in the number of channels showed that only channels in the IR range, to the right of the “red-edge” point, remained in the seven channels of the training basis, i.e., with a wavelength greater than 700 nm.

The study revealed a number of limitations that we hope to overcome in the future. First and foremost is the insufficiently high resolution of plant leaf images relative to the spatial resolution of HSI (1.5 mm/pix) and, especially, TIR (2.4 mm/pix) images, which did not achievethe desired detail of the leaf (in our case, wheat) and could not confidently separate both leaf and soil, nor one leaf from another. Secondly, the procedure for minimizing the number of channels was not fully automated, and a global minimum for the number of HSI channels and the *RMSE* error was not found. Thirdly, due to the limitation of the illumination spectrum from above with a value of about 880 nm, a part of the range of the hyperspectrometer 880–1000 nm has not been studied (see Figure 1).

The limitations are not fundamental, and in continuation of the study, we plan to: (1) conduct an experiment with a significantly higher HSI and TIR resolution for a plant leaf; (2) to automate the search for the global minimum of the number of channels carrying HSI knowledge about TIR for a given *RMSE* error, both based on plant biophysics and existing progressive methods, including [30,31]; and (3) expand both the research range in NIR up to 1000 nm and the functions of the R-XAI research model as a whole.

## Figures and Tables

**Table 1:** Key days numbers and plant pots numbers included in the ‘Train’ and ‘Test’ dataset parts.

Key days number	1	3	6	8	12	19	25
‘Train’ pots numbers	5	11	9	5	10	5	10
‘Test’ pots numbers	8	8	8	8	8	4	8

**Table 2:** Examples of channel values and *RMSE* that were achieved for two thresholds and different values of *a*,*b*. The best values for each day are highlighted in the background.

Day	Threshold	*a*,*b*	Mask, Pix	PLCC	Best Ch., No/nm	*RMSE*, °C
1	0.4	1	1274	0.616	58/566	0.159
1	0.5	1	867	0.57	144/823	0.151
1	0.5	0.1	483	0.827	143/820	0.056
3	0.4	1	1134	0.325	55/557	0.207
3	0.5	1	700	0.277	52/549	0.194
3	0.5	0.1	349	0.48	143/820	0.067
6	0.4	1	1232	0.464	53/551	0.212
6	0.5	1	824	0.41	118/745	0.214
6	0.5	0.1	337	0.685	143/820	0.058
8	0.4	1	1376	0.596	117/742	0.212
8	0.5	1	952	0.496	117/742	0.205
8	0.5	0.15	590	0.774	143/820	0.085
12	0.4	1	1954	0.576	142/817	0.198
12	0.5	1	1495	0.537	141/814	0.181
12	0.5	0.1	662	0.836	143/820	0.06
19	0.4	1	1124	−0.465	145/826	0.306
19	0.5	1	768	−0.414	145/826	0.264
19	0.4	0.1	482	−0.795	143/820	0.18
25	0.4	1	974	−0.434	128/775	0.364
25	0.4	0.1	398	−0.815	129/778	0.184
25	0.4	0.05	166	−0.957	143/820	0.189
25	0.5	1	588	−0.384	143/820	0.371
25	0.5	0.1	171	−0.828	58/566	0.189

**Table 3:** The best accuracies that were achieved using Formula (3) for the 143rd channel at thresholds of 0.4, 0.5, and small values of *a*,*b* compared to the area of the threshold mask (at *a*,*b* = 1) and 2 other masks: ‘in’ and ‘out’ of small *a*,*b* mask. For a full table for the entire threshold range, see Table A2.

Day	Threshold	PLCC *a*,*b* = 1	RMSE *a*,*b* = 1, °C	*a*,*b*	Mask in, Pix	PLCC in	*RMSE* in, °C	*RMSE* out, °C
1	0.4	0.60	0.18	0.08	556	0.91	0.06	0.23
1	0.5	0.56	0.17	0.06	316	0.92	0.05	0.2
3	0.4	0.18	0.29	0.05	1134	0.18	0.29	0.32
3	0.5	0.17	0.26	0.04	700	0.17	0.26	0.29
6	0.4	0.35	0.31	0.05	255	0.95	0.17	0.34
6	0.5	0.26	0.32	0.04	166	0.9	0.17	0.34
8	0.4	0.57	0.26	0.1	1376	0.57	0.26	0.32
8	0.5	0.49	0.26	0.1	952	0.49	0.26	0.33
12	0.4	0.52	0.24	0.1	1954	0.52	0.24	0.3
12	0.5	0.47	0.21	0.1	1495	0.47	0.21	0.27
19	0.4	−0.43	0.27	0.05	237	−0.95	0.15	0.29
19	0.5	−0.37	0.25	0.05	175	−0.91	0.14	0.27
25	0.4	−0.42	0.32	0.02	49	−1	0.11	0.33
25	0.5	−0.38	0.33	0.01	17	−1	0.11	0.33

**Table 4:** The influence of the parameters {Threshold; *a*,*b*} on both the temperature prediction accuracy in *RMSE* values and the Mask area for Train and Test datasets.

Threshold	*a = b*	TrainMask pix	TestMask pix	Train *RMSE*, °C	Test *RMSE*, °C	Epoch	Time, Sec
0.1	1	16,541	17,660	0.23	0.22	112	44
0.1	0.5	16,121	17,333	0.21	0.20	97	37
0.1	0.1	6870	7227	0.15	0.11	188	31
0.2	1	13,636	14,861	0.26	0.21	236	77
0.2	0.5	13,333	14,633	0.20	0.20	209	68
0.2	0.1	5769	6207	0.15	0.11	200	29
0.3	1	11,146	12,067	0.22	0.20	146	39
0.3	0.5	10,938	11,920	0.21	0.19	79	20
0.3	0.1	4740	5137	0.12	0.11	113	13
0.4	1	8581	9068	0.20	0.20	221	45
0.4	0.5	8465	8985	0.19	0.19	254	52
0.4	0.1	3777	3938	0.09	0.10	225	12
0.5	1	6126	6194	0.19	0.19	106	16
0.5	0.5	6074	6164	0.20	0.18	252	153
0.5	0.1	2762	2828	0.10	0.11	266	41

**Table 5:** Study of the masks area and the *RMSE* of temperature prediction for a number of channel groups in the parameters {number of channels (*k*); threshold; *a*,*b*}.

Number of Channels	Threshold	*a* = *b*	Train Mask, pix	Test Mask, pix	Train *RMSE*, °C	Test *RMSE*, °C	Epoch	Time, Sec
204	0.1	0.5	16,121	17,333	0.21	0.34	48	35.35
204	0.1	0.1	6870	7227	0.14	0.27	99	34.1
204	0.3	0.5	10,938	11,920	0.21	0.28	61	37.64
204	0.3	0.1	4740	5137	0.11	0.21	238	61.41
204	0.4	0.5	8465	8985	0.17	0.27	120	66.75
204	0.4	0.1	3777	3938	0.10	0.21	316	57.65
204	0.5	0.5	6074	6164	0.15	0.25	105	48.57
204	0.5	0.1	2762	2828	0.08	0.24	266	46.39
146 (15–160)	0.5	0.5	6074	6164	0.16	0.20	294	98.05
146 (15–160)	0.5	0.1	2762	2828	0.11	0.14	278	33.17
32 *	0.5	0.5	6074	6164	0.21	0.24	132	19.19
32 *	0.5	0.1	2762	2828	0.16	0.19	207	13.84
8 **	0.5	0.5	6074	6164	0.24	0.27	279	53.31
8 **	0.5	0.1	2762	2828	0.21	0.22	151	13.88
7 **	0.5	0.5	6074	6164	0.26	0.27	214	58.12
7 **	0.5	0.1	2762	2828	0.22	0.22	181	15.22

32 *—32 channels {9, 12, 14, 22, 25, 28, 36, 47, 56, 59, 71, 75, 82, 85, 88, 90, 95, 108, 111, 116, 120, 122, 126, 131, 133, 136, 139, 143, 150, 153, 157, 160} selected as PLCC curve extrema. 8 **—8 channels {95, 116, 126, 136, 143, 153, 157, 160} selected as mixture of PLCC extrema and channels popular in agriculture (highlighted by bold font further). Their wavelengths (nm) {**675**, **740**, 768, **800**, 820, **850**, 862, 870}. 7 **—7 channels {116, 126, 136, 143, 153, 157, 160} selected as for 8 ** without 95th channel.

## Data Availability

The data used here of a 25-day experiment on wheat drought with fixation of the state of plants in the control and experimental groups every 2–3 days using three types of sensors (HSI, Thermal IR, RGB) occupy 72.2 GB. The data can be obtained for free use upon request.

## Appendix A

**Table A1:** Investigation of possibilities for temperature prediction via Formula (3) based on the 143rd HSI channel (820 nm) on the example of Train dataset in the values of the Mask, PLCC, as well as *RMSE* and the parameters: key day; threshold value (*thr* = 0.1–0.5); *a = b =* 1. The maximum area for plants’ mask together with soil is 3136 pixels.

Day	Threshold	Mask, pix	PLCC	*RMSE*, °C	TIR Max	TIR Min	TIR Mean	TIR Std	Chan. Max	Chan. Min	Chan. Mean	Chan. Std
1	0.1	2534	0.75	0.21	21.5	20.2	20.8	0.23	1.37	0.05	0.53	0.29
1	0.2	2159	0.70	0.19	21.5	20.3	20.9	0.21	1.37	0.05	0.59	0.27
1	0.3	1702	0.63	0.19	21.5	20.4	20.9	0.19	1.37	0.16	0.67	0.24
1	0.4	1274	0.60	0.18	21.5	20.5	21.0	0.18	1.37	0.21	0.74	0.21
1	0.5	867	0.56	0.17	21.5	20.5	21.0	0.16	1.37	0.32	0.82	0.19
3	0.1	2744	0.52	0.28	21.5	20.1	20.9	0.23	1.42	0.05	0.49	0.30
3	0.2	2156	0.41	0.28	21.5	20.1	20.9	0.21	1.42	0.05	0.58	0.27
3	0.3	1643	0.29	0.29	21.5	20.1	21.0	0.19	1.42	0.16	0.67	0.24
3	0.4	1134	0.18	0.29	21.5	20.3	21.0	0.18	1.42	0.26	0.77	0.21
3	0.5	700	0.17	0.26	21.5	20.5	21.0	0.16	1.42	0.42	0.86	0.19
6	0.1	2411	0.52	0.32	21.6	20.1	20.9	0.24	1.30	0.04	0.57	0.28
6	0.2	2024	0.45	0.33	21.6	20.1	20.9	0.23	1.30	0.07	0.64	0.25
6	0.3	1633	0.40	0.32	21.6	20.1	20.9	0.21	1.30	0.11	0.71	0.22
6	0.4	1232	0.35	0.31	21.6	20.3	21.0	0.21	1.30	0.22	0.77	0.20
6	0.5	824	0.26	0.32	21.6	20.3	21.0	0.20	1.30	0.33	0.85	0.17
8	0.1	2492	0.68	0.30	20.9	19.1	20.1	0.30	1.37	0.04	0.62	0.28
8	0.2	2114	0.63	0.29	20.9	19.3	20.1	0.27	1.37	0.12	0.69	0.24
8	0.3	1761	0.59	0.28	20.9	19.4	20.2	0.25	1.37	0.19	0.75	0.21
8	0.4	1376	0.57	0.26	20.9	19.5	20.2	0.24	1.37	0.31	0.81	0.19
8	0.5	952	0.49	0.26	20.9	19.7	20.3	0.20	1.37	0.38	0.88	0.16
12	0.1	2823	0.65	0.30	21.4	19.6	20.6	0.30	1.42	0.08	0.70	0.28
12	0.2	2626	0.61	0.28	21.4	19.8	20.7	0.27	1.42	0.12	0.73	0.25
12	0.3	2359	0.57	0.26	21.4	19.9	20.7	0.25	1.42	0.20	0.77	0.22
12	0.4	1954	0.52	0.24	21.4	20.1	20.8	0.21	1.42	0.20	0.83	0.19
12	0.5	1495	0.47	0.21	21.3	20.2	20.8	0.19	1.42	0.40	0.89	0.17
19	0.1	2367	−0.54	0.33	22.2	21.2	21.7	0.22	1.59	0.09	0.65	0.30
19	0.2	1940	−0.49	0.31	22.2	21.2	21.6	0.21	1.59	0.23	0.73	0.27
19	0.3	1546	−0.49	0.30	22.2	21.2	21.6	0.20	1.59	0.32	0.81	0.25
19	0.4	1124	−0.43	0.27	22.2	21.2	21.6	0.18	1.59	0.41	0.91	0.22
19	0.5	768	−0.37	0.25	22.0	21.2	21.5	0.16	1.59	0.59	1.00	0.19
25	0.1	2289	−0.54	0.35	22.6	21.2	22.0	0.22	1.94	0.17	0.89	0.34
25	0.2	1842	−0.50	0.34	22.5	21.2	22.0	0.22	1.94	0.28	0.99	0.30
25	0.3	1423	−0.48	0.33	22.5	21.2	21.9	0.22	1.94	0.39	1.08	0.26
25	0.4	974	−0.42	0.32	22.4	21.2	21.9	0.21	1.94	0.50	1.19	0.23
25	0.5	588	−0.38	0.33	22.4	21.2	21.8	0.22	1.94	0.72	1.30	0.20

**Table A2:** Investigation of possibilities for temperature prediction via Formula (3) based on the 143rd HSI channel (820 nm) at all thresholds in values *RMSE*, and PLCC with *a* = *b* = 1 compared with PLCC and *RMSE* values inside a Mask ‘in’ truncated by reduced values of *a* and *b*, and *RMSE* for a mask ‘out’ of the Mask ‘in’, but in the full Mask (Table A1). The values are shown for each key day. The best values are highlighted with a light gray background. ‘Out’ values are 1.5 to 4 times larger than ‘in’.

Day	Threshold	PLCC *a*,*b* = 1	*RMSE* *a*,*b* = 1, °C	*a = b*	Mask in, pix	PLCC in	*RMSE* in, °C	*RMSE* out, °C	TIR in Max	TIR in Min	TIR in Mean	TIR in Std
1	0.1	0.75	0.21	0.1	1243	0.95	0.14	0.26	21.38	20.45	20.82	0.17
1	0.2	0.70	0.19	0.1	1110	0.93	0.09	0.26	21.25	20.49	20.85	0.15
1	0.3	0.63	0.19	0.1	888	0.9	0.08	0.26	21.25	20.58	20.90	0.13
1	0.4	0.60	0.18	0.08	556	0.91	0.06	0.23	21.25	20.65	20.95	0.11
1	0.5	0.56	0.17	0.06	316	0.92	0.05	0.2	21.23	20.77	20.99	0.09
3	0.1	0.52	0.28	0.1	1207	0.92	0.19	0.35	21.50	20.07	20.87	0.23
3	0.2	0.41	0.28	0.08	735	0.9	0.2	0.32	21.50	20.10	20.92	0.21
3	0.3	0.29	0.29	0.06	447	0.82	0.2	0.32	21.50	20.10	20.96	0.19
3	0.4	0.18	0.29	0.05	284	0.64	0.2	0.32	21.50	20.31	21.00	0.18
3	0.5	0.17	0.26	0.04	141	0.63	0.17	0.29	21.49	20.52	21.04	0.16
6	0.1	0.52	0.32	0.08	730	0.94	0.16	0.36	21.23	20.58	20.88	0.13
6	0.2	0.45	0.33	0.07	578	0.93	0.17	0.37	21.18	20.62	20.91	0.11
6	0.3	0.40	0.32	0.05	353	0.95	0.17	0.35	21.18	20.73	20.94	0.09
6	0.4	0.35	0.31	0.05	255	0.95	0.17	0.34	21.18	20.77	20.99	0.08
6	0.5	0.26	0.32	0.04	166	0.9	0.17	0.34	21.18	20.89	21.03	0.05
8	0.1	0.68	0.30	0.1	887	0.96	0.15	0.36	20.88	19.14	20.05	0.30
8	0.2	0.63	0.29	0.1	782	0.95	0.13	0.36	20.88	19.29	20.11	0.27
8	0.3	0.59	0.28	0.1	675	0.94	0.13	0.34	20.88	19.39	20.16	0.25
8	0.4	0.57	0.26	0.1	559	0.92	0.13	0.32	20.88	19.54	20.22	0.24
8	0.5	0.49	0.26	0.1	430	0.88	0.15	0.33	20.88	19.69	20.29	0.20
12	0.1	0.65	0.30	0.1	1063	0.96	0.14	0.37	21.35	19.62	20.64	0.30
12	0.2	0.61	0.28	0.1	1003	0.95	0.12	0.35	21.35	19.76	20.68	0.27
12	0.3	0.57	0.26	0.1	921	0.93	0.11	0.33	21.35	19.85	20.71	0.25
12	0.4	0.52	0.24	0.1	794	0.89	0.09	0.3	21.35	20.09	20.76	0.21
12	0.5	0.47	0.21	0.1	662	0.84	0.08	0.27	21.33	20.19	20.81	0.19
19	0.1	−0.54	0.33	0.04	359	−0.99	0.21	0.35	21.86	21.33	21.63	0.13
19	0.2	−0.49	0.31	0.04	302	−0.98	0.18	0.33	21.81	21.33	21.60	0.11
19	0.3	−0.49	0.30	0.04	244	−0.98	0.17	0.32	21.76	21.33	21.57	0.11
19	0.4	−0.43	0.27	0.05	237	−0.95	0.15	0.29	21.73	21.33	21.53	0.09
19	0.5	−0.37	0.25	0.05	175	−0.91	0.14	0.27	21.65	21.33	21.49	0.07
25	0.1	−0.54	0.35	0.04	415	−0.98	0.17	0.37	22.23	21.61	22.01	0.10
25	0.2	−0.50	0.34	0.03	234	−0.98	0.14	0.35	22.17	21.61	21.97	0.09
25	0.3	−0.48	0.33	0.03	166	−0.99	0.13	0.34	22.13	21.61	21.95	0.09
25	0.4	−0.42	0.32	0.02	49	−1	0.11	0.33	22.13	21.73	21.90	0.09
25	0.5	−0.38	0.33	0.01	17	−1	0.11	0.33	21.96	21.67	21.83	0.09
